# Adjustable Propagation Length Enhancement of the Surface Plasmon Polariton Wave via Phase Sensitive Optical Parametric Amplification

**DOI:** 10.1038/s41598-018-33831-y

**Published:** 2018-10-19

**Authors:** Mohammad Amin Izadi, Rahman Nouroozi

**Affiliations:** Department of Physics, Institute for Advanced Studies in Basic Sciences, Zanjan, 45137-66731 Iran

## Abstract

The adjustable propagation length enhancement of the surface plasmon polariton (SPP) mode under the effects of the initial relative phase (*ψ*_0_) between interacting waves in difference frequency generation (DFG) based optical parametric amplification (OPA) are numerically considered. The waveguide is a silver coated PPLN planar waveguide. Obtained results indicate ultra long propagation length for the SPP mode could be achieved via manipulation of *ψ*_0_ in exact quasi phase matching (QPM) case up to 30 mm for initial pump intensity about 66 MW/cm for degenerate DFG (dDFG). For chirped QPM by mitigating the high depletion of the pump intensity, it is possible to enhance the SPP propagation length up to 43 mm for initial pump intensity about 135 MW/cm. In this case *ψ*_0_ does not affect the SPP propagation length except around a narrow range of unsuitable phases. The unsuitable phase is $\frac{\pi }{2}$ for exact QPM but is pump dependent for chirped QPM case. Using this unsuitable phase is the key parameter to the SPP propagation length enhancement via controlling *ψ*_0_. In this case with a high pump intensity, the pump and the SPP modes interact at longer distances which leads to the SPP propagation length enhancement.

## Introduction

In recent years, devices which use surface plasmon polaritons (SPPs) have become the subject of interest in nano photonics^1^. This is due to miniaturizing of optical circuits for manipulating of light on sub-wavelength dimensions. Also, besides transferring of wide band optical signals using SPP waveguides, electronic data can be transfered because of using metal strips. By definition, the SPP is an electromagnetic mode which is generated when a TM optical wave couples with surface plasmons of noble metals. This coupling causes a coherent oscillation of the metal gas in close proximity of the metal surface which propagates along the interface between the noble metal and the dielectric^2^. Therefore, the SPP mode is a specific electromagnetic wave that the peak of its electromagnetic energy locates at the interface and decays exponentially in the perpendicular direction to the propagation direction. The decaying length from the metal side is on the order of 20 nm at optical frequencies^2^. As one can see, this small transversal dimension is completely below the diffraction limit and is the unique and marvelous property of SPPs which is not possible in conventional dielectric waveguides. Therefore, in the near future, the optical devices which use wide band optical frequencies to transfer data with sizes as small as nano electronic devices are feasible. Despite the positive points mentioned, strong interaction of light wave with surface plasmons leads to a fast dissipation of the electromagnetic energy in the lengths as short as few hundred micrometers^3^. This very short propagation length at optical frequencies, is the main obstacle to emergence of practical SPP based nano photonic devices. In order to have a practical SPP based photonic devices, the SPP propagation lengths should be in the order of at least few centimeters^4^. This causes a widespread theoretical and experimental studies to enhance the SPP propagation length. In THz regime, the SPP-like surface mode can propagate up to about a centimeter, but the mode confinement is much larger than the diffraction limit and the mode is very similar to the grazing surface mode^2^. Generally, the methods of enhancement of the SPP propagation length are divided into two major groups in the optical range: 1- methods which relay on linear amplification and propagation length enhancement of SPPs, like using gain media which induces extra noises^5,6^ (the enhanced SPP propagation length is about 1 cm in this case), utilizing long range SPPs which enhance the SPP propagation length up to some millimeters^7,8^, regenerating of SPP waves in an asymmetric waveguide structure^9^ and enhancement of surface plasmon polariton propagation length by the interference with photonic modes^10^. 2- methods which use nonlinear interactions between a hybrid guided mode (HGM) and SPPs^11^. Nonlinear interactions which involve SPP waves have attracted many attentions due to the strong localization and low group velocity of SPPs^12^. These could lead to strong nonlinear interactions and enhancement of the interaction time^13^. The nonlinear interaction to amplify and enhance the propagation length of SPPs is difference frequency generation (DFG) in a *χ*^(2)^ nonlinear substrate^14^. Among a wide range of *χ*^(2)^-based nonlinear optical materials, lithium niobate (LN) is the most widely used substrate to study DFG-based optical parametric amplification (OPA)^15,16^. Some prominent properties of LN include: a) having a large second order nonlinear coefficient (*d*_33_), b) transparency over a wide range of frequencies around communication band, c) ferroelectric behavior, d) fast optical response, e) small absorption coefficient and f) low spontaneous emission noise. The ferroelectric behavior enables us to create ferroelectric domain inverted LN or periodically poled LN (PPLN) to compensate the phase mismatch between wave vectors of the interacting waves^17^. This method is called quasi phase matching (QPM) technique.

In a DFG-OPA through a *χ*^(2)^-based nonlinear optical medium, a strong pump wave with a higher angular frequency (*ω*_*p*_) interacts with a weak signal wave of angular frequency *ω*_*s*_. During this interaction, besides the amplification of the signal wave, an idler wave with angular frequency *ω*_*i*_ = *ω*_*p*_ − *ω*_*s*_ is generated. From quantum mechanical point of view, with the annihilation of one higher energy pump photon, one signal photon and one idler photon are generated ($\hslash {\omega }_{p}=\hslash {\omega }_{s}+\hslash {\omega }_{i}$). Because of the difference between *ω*_*s*_ and *ω*_*i*_, the usual DFG interaction is sometimes called non-degenerate DFG (ndDFG).

It was shown that the initial relative phase between pump and signal (*ψ*_0_) in DFG-OPA interactions may affect the rate of signal amplification and idler generation^18,19^. According to this sensitivity to *ψ*_0_, DFG-OPA processes can be categorized into two major classes:

In a phase insensitive (PIS) OPA, which occurs in the usual ndDFG interaction, the SPP idler generation, SPP signal/idler amplification and SPP signal/idler propagation length enhancement does not depend on *ψ*_0_. This type of amplification, except the noise figure, is similar to erbium doped and semiconductor amplifiers. Therefore they are limited to quantum noise^20^. A special case of DFG interaction in which, the pump frequency (*ω*_*p*_) is exactly two times the signal frequency (*ω*_*s*_), with the annihilation of one pump photon, two signal photons are generated. This type of DFG interaction is called degenerate-DFG (dDFG) and we have ($\hslash {\omega }_{p}=\hslash {\omega }_{s}+\hslash {\omega }_{s}$). The dDFG process in second order nonlinear (*χ*^(2)^) substrates is the main method to make phase sensitive (PS) amplification of the signal, where *ψ*_0_ affects the rate of the SPP amplification and SPP length enhancement. In this case, the noise figure is substantially below the 3 dB quantum limit of the PIS interactions^18^. An interesting and easy way to turn PIS-ndDFG into the PS-ndDFG is to inject a wave with angular frequency of *ω*_*i*_ at the beginning of interaction (waveguide input).

In previous reports^21,22^, propagation length enhancement of a SPP wave along a silver coated PPLN planar waveguide (it will be called “waveguide” in the rest of this paper) was investigated via *χ*^2^-based DFG interaction. The nonlinear interaction occurs between a HGM and SPP modes for two different types of QPM domain grating. First^21^, the grating wavelength (Λ) was considered constant in such a way that it compensates the phase mismatch between the HGM and SPP modes exactly (therefore, it is called exact QPM (EQPM) where its wavelength is ${{\rm{\Lambda }}}_{e}=\frac{2\pi }{{\rm{\Delta }}\beta }$ and Δ*β* is the phase mismatch between pump, signal and idler). It was shown that an increased pump intensity above the threshold level leads to a strong interaction between the HGM pump and the SPP signal/idler for the ndDFG process. Consequently, the HGM pump depletes in a shorter propagation length and cannot provide enough power to compensate the SPP modes dissipation, despite the high amplification of SPP modes in the shorter lengths. To suppress the strong interaction between the HGM pump and the SPP signal/idler, an inverse linearly chirped nonlinear grating with wavelength (${\rm{\Lambda }}=\frac{{{\rm{\Lambda }}}_{0}}{1+rx}$, where Λ_0_ is the wavelength at the beginning of the waveguide and *r* is the chirping rate) was proposed^22^ in the second report. In this type of chirped QPM (CQPM) method, the wavelength (Λ) of the domain inverted grating varies along the waveguide. At the input side it differs from the exact value of the EQPM condition (Λ_*e*_) and it changes slightly to the (Λ_*e*_) along the waveguide. This indeed causes a relative phase mismatch between interacting modes. Thus the strong interaction can be mitigated in a phase sensitive manner. Since the high amplification of the SPP modes is not seen in shorter propagation lengths, the HGM pump has enough power for the non-phase-matched amplification of the SPP modes. Therefore longer propagation lengths of the SPP signal/idler was achieved up to 43 mm. This enhanced SPP propagation length is the most SPP propagation length to the best of our knowledge using one of the methods of phase sensitivity. Therefore the main purpose of these published studies is only propagation length enhancement of the SPP modes in the optical range.

The purpose of this paper is to describe comprehensively all PIS and PS DFG-based adjustable propagation length enhancement and amplification of the SPP waves along the proposed waveguide. Therefore, in what follows, the proposed waveguide will be introduced, the profile of the real part of the electric fields for HGM mode and SPP modes are illustrated and the dispersion relation for each modes are discussed. The main body of the paper is the results section. In this section, the phase sensitivity in all types of DFG based SPP propagation length enhancement and amplification are investigated in detail. To reach this goal, coupled mode equations for describing DFG processes are solved numerically^15^ in the slowly varying envelope (SVE) approximation and the results are discussed. According to the results, any SPP propagation length could be achieved via adjusting the initial relative phase between interacting waves on one hand and the pump power on the other hand. Two distinct cases have been considered:

- PIS case in ndDFG interaction for EQPM condition.
- PS interactions and related methods to achieve them.

## Proposed Waveguide and its Specifications

**Figure 1:**
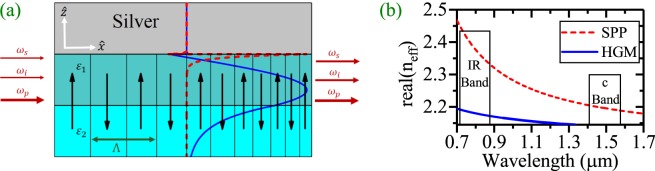
(**a**) The schematic illustration of a silver coated three layer inverse linearly chirped planar waveguide with the core thickness = 2 *μ*m. *ε*_1_ and *ε*_2_ are the permittivity tensor of the LN core and substrate at room temperature respectively, where *ε*_1_ = *ε*_2_ + 0.04. Each thick black arrow shows the direction of the domain inverted LN with duty cycle of 0.5. The PPLN can be exact or chirped according to the text (Λ is the wavelength of the nonlinear QPM grating). In this figure, Normalized real part of the transverse electric field (*E*_*z*_) distributions of the SPP mode (red-dashed line) and the HGM (blue-solid line) in the waveguide are presented. The maximum of the electric field for the HGM (blue-solid line) is in the dielectric PPLN while that of the SPP mode (red-dashed line) is located at the metal-dielectric interface. (**b**) Dispersion relation of the excited modes in the waveguide. In the c-band only the SPP mode can be excited and no guided mode is present. The HGM mode in the IR range (775 nm) is used to pump the parametric amplification of the SPP modes in the c-band.

The proposed waveguide in this paper, is a silver coated PPLN planar waveguide. The difference between refractive indices of the central layer with respect to the substrate and the thickness of the central layer determine the dispersion properties of the waveguide (how many modes the waveguide can support; see Fig. 1(a)). In this study, lithium niobate is considered as an anisotropic medium and the optical axis is parallel to the z-direction^23^. x-direction is the propagation direction. *ε*_2_ is the permittivity tensor of the lithium niobate for substrate and *ε*_1_ as the permittivity tensor of the core of the waveguide. To have only two modes, the thickness of the core should be 2 *μm* for *ε*_1_ = *ε*_2_ + 0.04. This difference in permittivity tensor corresponds to a refractive index change of about 10^−3^ and can be realized via Ti in-diffusion method^15^.

Also, Fig. 1(a) illustrates the distribution of the real part of the transversal electric fields (*E*_*z*_) of the HGM (blue-solid line) and the SPP mode (red-dashed line). From this figure it is inferred that, the peak of the electric field strength for the SPP mode locates at the metal-dielectric interface. This causes a high interaction between the electric field and the surface plasmons and a strong absorption of the electromagnetic energy. On the other hand, the peak of the electric field strength for the HGM is away from the metal-dielectric interface. The dispersion relation for the waveguide is depicted in Fig. 1(b). One can see that for the reported values of the thickness, *ε*_1_ and *ε*_2_, the SPP modes exists for a vast range of wavelengths from 700 nm to 1700 nm. But the waveguide only can support the HGM for wavelengths shorter than 1350 nm.

Table 1 presents the effective refractive indices of the HGM (pump) and the SPP modes (signal/idler) at 775 nm and 1540, 1550 and 1560.13 nm in the waveguide, respectively. The calculations show that the attenuation for the HGM mode is 1000 times less than that of the SPP modes. This is because of the weak interaction between the electric field of the HGM mode with the metal compared to the strong interaction between the electric field of the SPP mode with the free electrons of the metal surface.

**Table 1:** Effective refractive indices of the allowed modes in the waveguide.

Mode Type	Wavelength	Real (*n*_*eff*_)	Im (*n*_*eff*_)
HGM	775 nm	2.1830	2.861 × 10^−6^
SPP mode	1540 nm	2.1915	1 × 10^−3^
SPP mode	1550 nm	2.1906	1 × 10^−3^
SPP mode	1560.13 nm	2.1897	1 × 10^−3^

## Results

### Propagation length enhancement via phase insensitive DFG-based OPA

In this subsection propagation length enhancement via ndDFG-based OPA is presented briefly^21^. If the following complex notation for the electric field is assumed:

1 $$\begin{array}{l}{E}_{k}(x,z)={A}_{k}(x){E}_{k}^{0}(z){e}^{j[({\beta }_{k}+j\frac{{\alpha }_{k}}{2})]x},\end{array}$$

where ${E}_{k}^{0}(z)$ is the normalized transverse electric field distribution of the interacting waves, *β*_*k*_ and *α*_*k*_/2 are the real and imaginary parts of the wavevector, respectively. ${A}_{k}={a}_{k}{e}^{j({\varphi }_{k})}$ is the amplitude of the electric field of the interacting waves (with *a*_*k*_ and *ϕ*_*k*_ as their modulus and phase, respectively), the coupled mode equations describing the ndDFG-OPA interaction can be separated into the modulus and the phase parts as what fallows:

2 $$\begin{array}{rcl}\frac{d{a}_{p}}{dx} & = & -\frac{{\alpha }_{p}}{2}{a}_{p}+{\omega }_{p}{\kappa }_{0}{a}_{s}{a}_{i}sin(\psi ),\\ \frac{d{a}_{s}}{dx} & = & -\frac{{\alpha }_{s}}{2}{a}_{s}-{\omega }_{s}{\kappa }_{0}{a}_{p}{a}_{i}sin(\psi ),\\ \frac{d{a}_{i}}{dx} & = & -\frac{{\alpha }_{i}}{2}{a}_{i}-{\omega }_{i}{\kappa }_{0}{a}_{p}{a}_{s}sin(\psi )\\ \frac{d{\varphi }_{p}}{dx} & = & \frac{{\omega }_{p}{\kappa }_{0}{a}_{s}{a}_{i}cos(\psi )}{{a}_{p}},\\ \frac{d{\varphi }_{s}}{dx} & = & \frac{{\omega }_{s}{\kappa }_{0}{a}_{p}{a}_{i}cos(\psi )}{{a}_{s}},\\ \frac{d{\varphi }_{i}}{dx} & = & \frac{{\omega }_{i}{\kappa }_{0}{a}_{p}{a}_{s}cos(\psi )}{{a}_{i}},\end{array}$$

here *ψ* = *ϕ*_0_ + *ϕ*_*p*_ − *ϕ*_*s*_ − *ϕ*_*i*_ − Δ*βx* + Φ(*x*) is the relative phase along the waveguide. The complex nonlinear coupling coefficient is ($\kappa =\frac{{\varepsilon }_{0}}{2}{\int }_{-\infty }^{\infty }{d}_{eff}{E}_{p}^{0}{E}_{s}^{0\ast }{E}_{i}^{0\ast }dz$), where *κ*_0_ and *ϕ*_0_ are the modulus and phase of *κ*. The subscripts (*k* = *p, s* and *i*) stand for pump, signal and idler respectively. The phase mismatch between HGM pump, SPP signal and SPP idler is:

3 $${\rm{\Delta }}\beta ={\beta }_{i}+\beta s-\beta p$$

and ${d}_{eff}=\frac{2}{\pi }{d}_{nl}{e}^{i{\rm{\Phi }}}$ is the first term of the Fourier series of the PPLN nonlinear grating and *d*_*nl*_ = −27.2 pm/v^15^, is the nonlinear coefficient of LN. In introducing *ψ*, ${\rm{\Phi }}=\int K(x)dx$ is the resultant phase of the nonlinear PPLN grating, where $K(x)=\frac{2\pi }{{\rm{\Lambda }}}$ and Λ (two-headed green arrow in Fig. 1(a)) are the wavevector and the wavelength of the nonlinear grating respectively. Equations 2 are obtained under SVE approximation.

When Λ is chosen in such a way that the phase mismatch between interacting HGM pump and SPP modes is compensated completely, i.e. Λ = Λ_*e*_ (EQPM), the injected pump transfers its power to the SPP signal and idler from the beginning of the waveguide. Because of the creation of the idler as soon as the ndDFG starts, the process is phase insensitive (PIS). This means that the generation of the idler wave does not depend on *ψ*_0_ as the initial relative phase. In order to understand this process deeply, the ndDFG interaction between the HGPM pump (*λ*_*p*_ = 775 nm, *I*_*p*,0_ = 31 MW/cm) and the SPP signal (*λ*_*s*_ = 1540 nm, *I*_*s*,0_ = 1 kW/cm) to generate a SPP idler (*λ*_*i*_ = 1560.13 nm) is investigated for EQPM case (in this paper *I*_*s*,0_ = 1 kW/cm for all cases). Λ_*e*_ in this case is 101.9 *μ*m^21^. Because of the complexity of the coupled nonlinear mode equations (2) described in this subsection, they cannot be solved analytically and should be solved numerically. The well-known Runge-Kutta method was used via the ODE45 code in OCTAVE software^24^.

**Figure 2:**
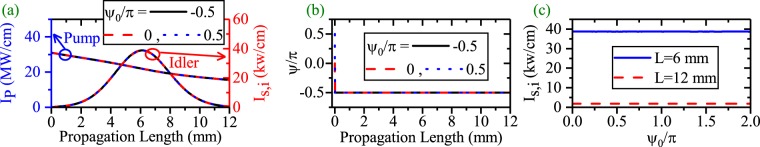
(**a**) Intensity variation of the HGM pump (left-blue vertical axis) and the SPP signal/idler generation and the SPP propagation length enhancement (right-red vertical axis) for ${\psi }_{0}=-\,\frac{\pi }{2},\,0$ and $\frac{\pi }{2}$. [*λ*_*p*_ = 775 nm, *λ*_*s*_ = 1540 nm, *λ*_*i*_ = 1560.13 nm]. (**b**) Relative phase (*ψ*) evolution along the waveguide for different *ψ*_0_ in ndDFG-EQPM case. (**c**) Effects of *ψ*_0_ on the generation of the SPP signal/idler at different length with respect to the different *ψ*_0_.

**Figure 3:**
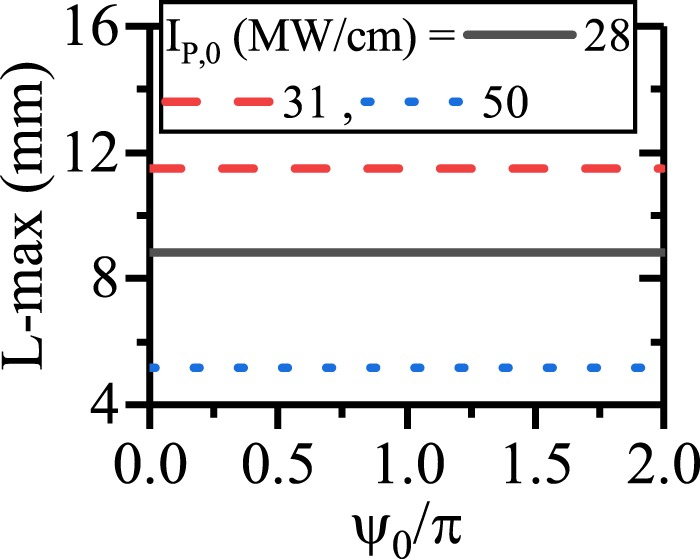
Maximum of the SPP signal/idler propagation length with respect to *ψ*_0_ for different values of *I*_*p*,0_ in ndDFG-EQPM waveguide.

Figure 2(a) shows the pump depletion and SPP signal/idler generation for different values of *ψ*_0_ (*λ*_*p*_ = 775 nm, *λ*_*s*_ = 1540 nm, *λ*_*i*_ = 1560.13 nm). The figure illustrates that *ψ*_0_ does not affect on the SPP idler generation when EQPM grating is used to compensate the phase mismatch between the interacting waves. The evolution of *ψ* along the waveguide is depicted in Fig. 2(b). Since during the EQPM-ndDFG process there is no phase degradations, the relative phase takes its stable value to generate the idler at the beginning of the waveguide to satisfy the sin(*ψ*) = −1 $(\psi =-\,\frac{\pi }{2})$ in equations 2. In this case, unidirectional transferring of power from the HGM pump to the SPP signal and idler occurs. Figure 2(b) also shows that *ψ* reaches to the stable value $(\psi =-\frac{\pi }{2})$ as soon as the ndDFG starts at beginning of the waveguide and does not change along it. As one can see in Fig. 2(c), the variation of *ψ*_0_ does not change the rate of SPP idler (therefore SPP signal) amplification at different lengths. This verifies the phase insensitivity of the ndDFG-OPA process for EQPM grating. Based on this process, the length enhancement up to 11.5 mm is achieved which is independent of the initial relative phase between interacting waves as it is shown in Fig. 3. In this figure, L-max denotes the length at which, the intensity of the SPP mode reaches 1 kw/cm after its amplification. As it is mentioned in the previous study^21^, the only prominent factor to enhance the SPP propagation length in this case is *I*_*p*,0_. For *I*_*p*,0_ = 31 MW/cm, the SPP propagation length reaches to 11.5 mm. Below this level of *I*_*p*,0_, the pump cannot amplify the SPP wave because of high attenuation of the SPP waves. Above this level of *I*_*p*,0_, the strong interaction between the interacting waves leads to a high depletion of the pump. In this case, the pump depletes fast and gives its power to the SPP waves in short distances. Consequently, higher values of *I*_*p*,0_ cannot lead to the SPP propagation length enhancement. This issue was discussed in the previous^21^ work comprehensively.

### Propagation length enhancement via phase sensitive DFG-based OPA

In order to induce phase sensitivity, the dDFG-OPA interaction^15^ has to be considered instead of ndDFG-OPA. According to Fig. 1(a), this happens when a HGM pump of angular frequency (*ω*_*p*_) and a signal of angular frequency $({\omega }_{s}=\frac{{\omega }_{p}}{2})$ are injected into the waveguide. Therefore when the process goes on, with the annihilation of one pump photon, two signal photons are generated resulting in a signal amplification (*λ*_*p*_ = 775 nm, *λ*_*s*_ = 1550 nm). There is a probability that the second harmonic wave of the signal interferes with the pump wave constructively which leads to a deamplification of the signal wave instead of its amplification. This occurrence strongly depends on the initial relative phase between the signal and pump waves (*ψ*_0_) at the beginning of the waveguide. Therefore the dDFG phenomenon is completely PS^15^. The dDFG-OPA interaction can also be analyzed in SVE approximation with a reduced version of equations 2:

4 $$\begin{array}{rcl}\frac{d{a}_{p}}{dx} & = & -\,\frac{{\alpha }_{p}}{2}{a}_{p}+{\omega }_{s}{\kappa }_{0}{a}_{s}^{2}sin(\psi ),\\ \frac{d{a}_{s}}{dx} & = & -\,\frac{{\alpha }_{s}}{2}{a}_{s}-{\omega }_{s}{\kappa }_{0}{a}_{p}{a}_{s}sin(\psi )\\ \frac{d{\varphi }_{p}}{dx} & = & \frac{{\omega }_{s}{\kappa }_{0}{a}_{s}^{2}cos(\psi )}{{a}_{p}},\\ \frac{d{\varphi }_{s}}{dx} & = & {\omega }_{s}{\kappa }_{0}{a}_{p}cos(\psi ),\end{array}$$

here, *ψ* =  *ϕ*_0_+ *ϕ*_*p*_ − 2*ϕ*_*s*_ − Δ*βx* + Φ(*x*) is the relative phase through the dDFG process. The complex nonlinear coupling coefficient is $\kappa =\frac{{\varepsilon }_{0}}{2}{\int }_{-\infty }^{\infty }{d}_{eff}{E}_{p}^{0}{({E}_{s}^{0\ast })}^{2}dz$, and the phase mismatch between the HGM pump and the SPP signal is:

5 $${\rm{\Delta }}\beta =2\beta s-\beta p.$$

**Figure 4:**
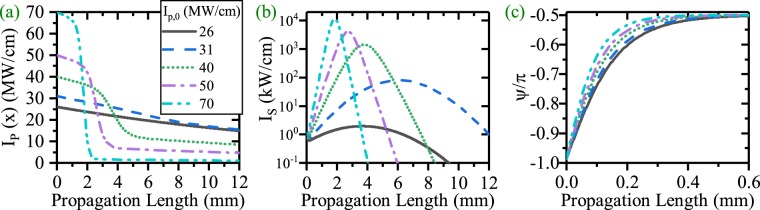
(**a**) Intensity variation of the HGM pump, (**b**) SPP signal amplification and SPP signal propagation length enhancement [*λ*_*p*_ = 775 nm, *λ*_*s*_ = 1550 nm]. (**c**) relative phase evolution (*ψ*) along the waveguide for different values of *I*_*p*,0_ in the dDFG-EQPM case.

When EQPM nonlinear grating is used, the Λ is chosen in such a way that the phase mismatch between the HGM pump and the SPP signal is compensated completely, i.e. ${\rm{\Lambda }}={{\rm{\Lambda }}}_{e}=\frac{2\pi }{{\rm{\Delta }}\beta }$. Panels (a), (b) and (c) of Fig. 4 depict the HGM pump variation, the SPP signal amplification (SPP propagation length enhancement) and the relative phase evolution (*ψ*) for the dDFG process respectively.

Since the threshold of the pump intensity for the SPP signal amplification is about 26 MW/cm^21^, As one can see, around the threshold level of the pump intensity, the interaction is weak and the pump does not deplete intensively. This manner continues to the pump intensity up to 31 MW/cm where the SPP signal has a peak at *x* = 6.06 mm with a signal amplification gain of about 19.06 dB. The maximum of the propagation length is achieved in this *I*_*p*,0_. Increasing *I*_*p*,0_ above this value of initial pump intensity leads to a strong interaction between the HGM pump and the SPP signal. In this case, pump depletes in a shorter length and cannot compensate the SPP attenuation in the rest of the waveguide. Thus for higher values of *I*_*p*,0_, besides the high amplification of the SPP signal, the propagation length of the SPP mode becomes shorter. These results are achieved when the initial phase of the HGM pump and the SPP signal (*ϕ*_*p*,0_, *ϕ*_*s*,0_) were considered zero.

The initial relative phase in Fig. 4(c) is the consequence of the complex value of *κ* and its role in the definition of *ψ*. Therefore it reaches to $-\frac{\pi }{2}$ in order to monotonic transferring the power from the HGM pump to the SPP signal. From the figure, it is inferred that, in the dDFG process, *ψ* evolves along the propagation direction until it reaches to the stable value of $-\frac{\pi }{2}$. According to equation 4, for creating unidirectional transferring power from the pump to the signal, sin(*ψ*) should be −1 or $\psi =-\,\frac{\pi }{2}$. The evolution of *ψ* depends on *I*_*p*,0_ in such a way that for higher values *I*_*p*,0_, *ψ* reaches to its stable value at shorter propagation lengths. This results in a strong amplification.

**Figure 5:**
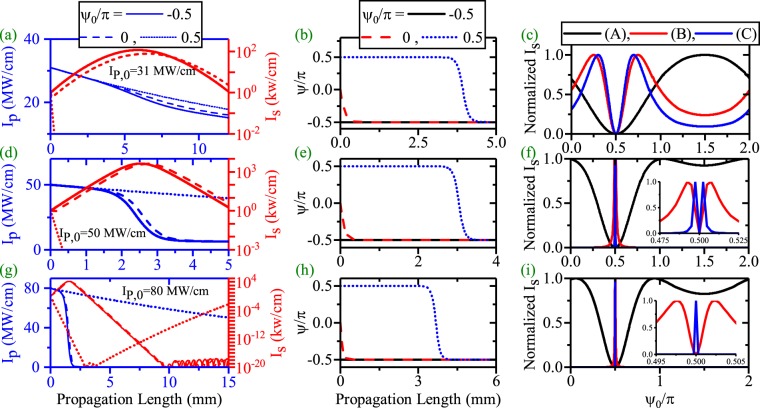
Effects of the *ψ*_0_ on the pump depletion, the SPP amplification and SPP length enhancement in dDFG-EQPM case. HGM pump depletion (blue) and SPP signal amplification and propagation length enhancement (red) along the propagation direction ((**a**),(**d**) and (**g**)). Relative phase (*ψ*) evolution along the propagation direction((**b**),(**e**) and (**h**)). Effects of *ψ*_0_ on the *I*_*s*_ at different position (phase sensitivity), [(**c**)-((A), L = 6.054 mm, *I*_*s*,*max*_ = 112 kw/cm), ((B), L = 12 mm, *I*_*s*,*max*_ = 1.87 kw/cm) and ((C), L = 14 mm, *I*_*s*,*max*_ = 0.18 kw/cm)], [(**f**)-((A), L = 2.703 mm, *I*_*s*,*max*_ = 4117 kw/cm), ((B), L = 2661 mm, *I*_*s*,*max*_ = 4117 kw/cm) and ((C), L = 10 mm, *I*_*s*,*max*_ = 0.08 kw/cm)], [(**i**)-((A), L = 1.67 mm, *I*_*s*,*max*_ = 15781 kw/cm), ((B), L = 3.21 mm, *I*_*s*,*max*_ = 13479 kw/cm) and ((C),L = 14 mm, *I*_*s*,*max*_ = 3418 kw/cm)].

Since *ψ* evolves along the waveguide, therefore *ψ*_0_ could affect the *ψ* evolution, hence the whole process (the SPP amplification and the SPP propagation length enhancement for EQPM case). This subject is completely investigated in Fig. 5. Panels (a),(d) and (g) of the figure illustrate the HGM pump (blue) and the SPP signal (red) variations for *I*_*p*,0_ = 31, 50 and 80 MW/cm and ${\psi }_{0}=-\,\frac{\pi }{2},0,\frac{\pi }{2}$ respectively. Clearly, *ψ*_0_ can affect the SPP amplification and SPP propagation length enhancement as what follows: for *ψ*_0_ = 0, the initial phase between pump and signal differs from the stable value of $-\frac{\pi }{2}$. Therefore, at the beginning of the waveguide, the pump can not amplify the SPP signal. Therefore, the SPP signal attenuates until *ψ* reaches the proper value according to Fig. 5(b). At this length, if the pump intensity is above the threshold level, amplification of the SPP signal starts. When ${\psi }_{0}=-\,\frac{\pi }{2}$, *ψ*_0_ and its stable value coincide with each other from the beginning. Therefore, the amplification of the SPP signal starts from the beginning of the waveguide and *ψ* does not change along the waveguide. According to panel (a), this initial phase leads to a higher amplification and a shorter propagation length. For ${\psi }_{0}=\frac{\pi }{2}$, the SPP signal gives its power to the HGM pump at the waveguide beginning as equations 4 rule it. Thus in this case the SPP signal deamplifies and pump amplifies until *ψ* reaches to the stable value. For *I*_*p*,0_ = 31 MW/cm, this happens around propagation length = 5 mm. At this length, the pump intensity is below the threshold level for amplification of the very weak SPP signal and cannot amplify it. These behaviors are also seen for *I*_*p*,0_ = 50 MW/cm. When, *I*_*p*,0_ = 80 MW/cm (panel(g)), again ${\psi }_{0}=-\frac{\pi }{2}$ results in a little stronger amplification of signal and a shorter propagation length with respect to *ψ*_0_ = 0. But for ${\psi }_{0}=\frac{\pi }{2}$, at the beginning, SPP signal attenuates to a very low level until *ψ* gets the proper value. At this length, the pump intensity is strong enough to interact with the very weak SPP signal and amplify it. Therefore, higher values of pump intensity is needed to enhance the SPP propagation length when ${\psi }_{0}=\frac{\pi }{2}$. In this case, the L-max of the SPP signal is about 20 mm. The phase sensitivity of the signal amplification at different propagation length are plotted in panel (c), (f) and (i) of Fig. 5. Measurements are done in the location of the peak intensity of the SPP signal (A), L-max (B) and in a place a bit more distant from the position of L-max (C) respectively when *ϕ*_*p*,0_ and *ϕ*_*s*,0_ are zero. To have a better vision, signal intensities are normalized with respect to its maximum values which are reported in the caption (*I*_*s*,*max*_). As one can see, for *I*_*p*,0_ = 31 MW/cm (low depletion limit), the signal amplification phase sensitivity is almost a sin function ((A)) where L = 6.054 mm and its minimum locates at ${\psi }_{0}=\frac{\pi }{2}$. This *ψ*_0_ does not lead to amplification of the SPP wave. For case (B) where L = 12 mm, this behavior differs from a sinusoidal pattern. Again the minimum locates at ${\psi }_{0}=\frac{\pi }{2}$. An enhancement of the measurement location (case (C)) where L = 14 mm, cannot amplify and enhance the SPP propagation length. This peculiar behavior of phase sensitivity where the PS amplification manner depends on the measurement location is not seen in all dielectric based dDFG waveguides^18^ where in the later case, the PS curve has almost a sinusoidal shape in all locations. When *I*_*p*,0_ = 50 MW/cm (panel (f)), the PS curve is completely different to a sinusoidal pattern even for peak position (case (A)) where L = 2.703 mm. Therefore, in the strong interaction regime, the amplification of the SPP wave at the peak position, almost phase insensitive except for *ψ*_0_ around $\frac{\pi }{2}$ where deamplification starts. At longer length (case (B) and (C)) where L = 5.34 mm and 10 mm respectively, the PS carves, almost have a delta shape except for ${\psi }_{0}=\frac{\pi }{2}$. For this *ψ*_0_, SPP amplification does not occur. The previous manner is also seen for *I*_*p*,0_ = 80 MW/cm in panel (i) for case (A) and (B) where L = 1.627 mm and 3.21 mm respectively. Because of the very high pump intensity, the SPP wave can be amplified even for ${\psi }_{0}=\frac{\pi }{2}$. As it can bee seen, for L = 14 mm (case (C)), the SPP signal is amplified which leads a SPP length enhancement.

**Figure 6:**
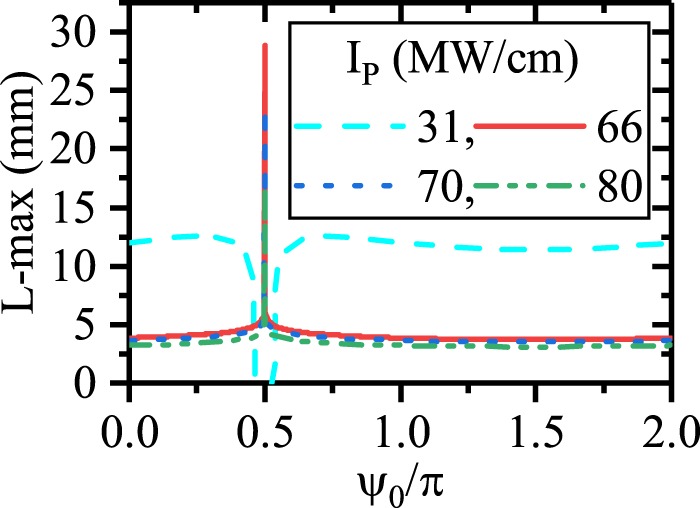
Maximum of the propagation length of SPP signal with respect to *ψ*_0_ for different values of *I*_*p*,0_ in dDFG-EQPM case.

To summarize the effect of *ψ*_0_ on the SPP propagation length enhancement in the dDFG interaction for EQPM case, the maximum of the propagation length (L-max) for different values of pump intensity with respect to *ψ*_0_ is depicted in Fig. 6.

From the figure, it is inferred that, to enhance the SPP propagation length via PS-dDFG interaction, *ψ*_0_ should be $\frac{\pi }{2}$ and the pump intensity should be high enough to interact and amplify the SPP signal at longer distances. For example, when *I*_*p*,0_ = 66 MW/cm, the maximum of SPP propagation length is about 30 mm. Again, higher values of *I*_*p*,0_ leads to a strong interaction in shorter distances. As it can be seen, *I*_*p*,0_ = 80 MW/cm results in a shorter SPP propagation length (17 mm).

The phase sensitive DFG-OPA is also possible via ndDFG. In this case a phase controllable light wave with angular frequency of *ω* = *ω*_*i*_ should be coupled together with the pump and the signal waves in the waveguide beginning (according to Fig. 1(a)). Then the sum frequency generation of signal and idler can interfere with the pump in a phase controllable manner. Therefore, the phase adjustment of the idler (*ψ*_0_) at the input leads to the amplification/deamplification of the signal/idler.

**Figure 7:**
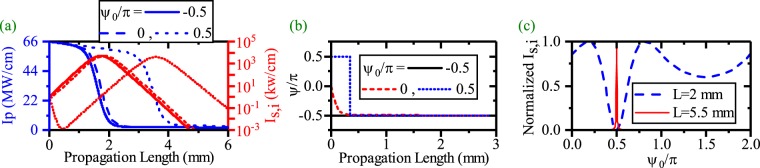
(**a**) Intensity variation of the HGM pump (left-blue vertical axis) and the SPP signal/idler generation and propagation length enhancement (right-red vertical axis) for ${\psi }_{0}=-\frac{\pi }{2}\mathrm{,0}$ and $\frac{\pi }{2}$, *I*_*p*,0_ = 66 MW/cm. (**b**) Relative phase (*ψ*) evolution along the waveguide for different *ψ*_0_ in ndDFG-EQPM case when a light wave of angular frequency *ω*_*i*_ is injected to the waveguide [*λ*_*p*_ = 775 nm, *λ*_*s*_ = 1540 nm, *λ*_*i*_ = 1560.13 nm]. (**c**) Effects of *ψ*_0_ on the generation of the SPP signal/idler at (L = 2 mm, *I*_*s*,*i*,*max*_ = 4923 kw/cm) and (L = 5.5 mm, *I*_*s*,*i*,*max*_ = 1 kw/cm), with respect to different *ψ*_0_.

Figure 7(a) shows the pump depletion, the SPP signal/idler generation and the SPP signal/idler propagation length enhancement in the ndDFG interaction where a light wave of angular frequency *ω*_*i*_ is injected at the beginning of the waveguide for *I*_*p*,0_ = 66 MW/cm and *I*_*i*,0_ = *I*_*s*,0_ (*λ*_*p*_ = 775 nm, *λ*_*s*_ = 1540 nm, *λ*_*i*_ = 1560.13 nm). With a comparison to Fig. 2(a), it becomes clear that *ψ*_0_ affects the SPP generation and the SPP propagation length enhancement when a light wave of angular frequency *ω*_*i*_ is injected to the waveguide. The results for ${\psi }_{0}=0,-\frac{\pi }{2}$ are the same as the results reported in Fig. 5(g). But when ${\psi }_{0}=\frac{\pi }{2}$, *ψ* needs less propagation length to achieve the stable value of $-\frac{\pi }{2}$ (see Fig. 7(b)). During this length, the SPP signal and the idler cannot be amplified. When *ψ* reaches $-\frac{\pi }{2}$, the SPP signal and idler start amplifying if the pump still has enough power to amplify them. Variation of the *ψ* along the propagation length is illustrated in Fig. 7(b) for different values of *ψ*_0_. It can be inferred that inserting a light wave of angular frequency *ω*_*i*_ changes the usual PIS-ndDFG into PS case. A comparison to Fig. 5(h) shows that inserting the idler wave at the waveguide beginning leads to a faster variation of *ψ*. This is because of an overall higher intensity for the SPP signal and idler. To have a better insight, the phase sensitivity of the signal/idler generation is investigated in Fig. 7(c) for *I*_*p*,0_ = 66 MW/cm at different measurement lengths. The results show that for L = 2 mm, the HGM pump has just begun to amplify the signal/idler SPP modes. Therefore there is a drop around ${\psi }_{0}=\frac{\pi }{2}$. In contrast, when L = 5.5 mm, because pump has enough energy to amplify signal/idler SPP modes, the SPP propagation length is enhanced. In this case the phase sensitivity has a delta shape.

**Figure 8:**
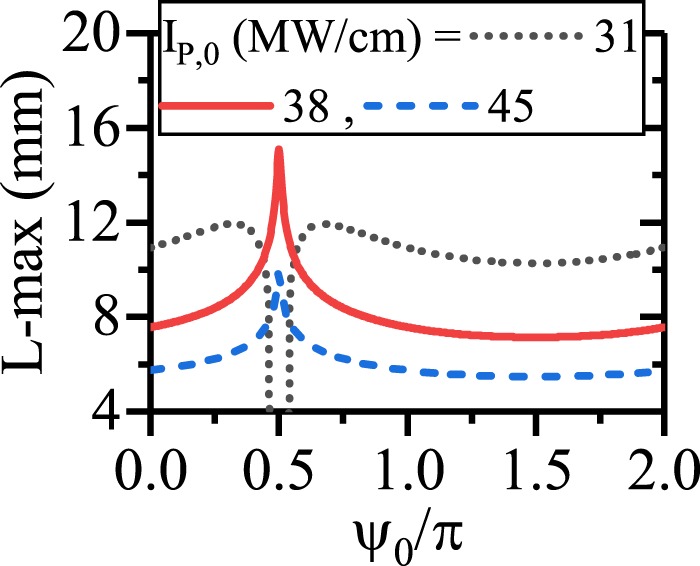
Maximum of the propagation length of the SPP signal/idler with respect to *ψ*_0_ for different values of *I*_*p*,0_ in ndDFG-EQPM case when a light wave of angular frequency *ω*_*i*_ is injected.

To give a finial result on the SPP propagation length enhancement in PS-ndDFG via inserting a light wave of angular frequency *ω*_*i*_ as the input, the effects of *ψ*_0_ on L-max are depicted in Fig. 8. As the figure illustrates, when *I*_*p*,0_ is around the threshold level (31 MW/cm), the pump does not have enough power to interact with SPP signal and idler for ${\psi }_{0}=\frac{\pi }{2}$. When *I*_*p*,0_ = 38 MW/cm, pump can enhance the propagation length for ${\psi }_{0}=\frac{\pi }{2}$ with enough power. In this case, L-max is 15.5 mm. Any enhancement of *I*_*p*,0_ results in a strong amplification and shortening the L-max. With a comparison to Fig. 6, it is inferred that, although inserting of a light wave of angular frequency *ω*_*i*_ as the input leads to a PS ndDFG, but the resultant L-max is shorter than that of the dDFG-PS length enhancement in EQPM waveguide.

**Figure 9:**
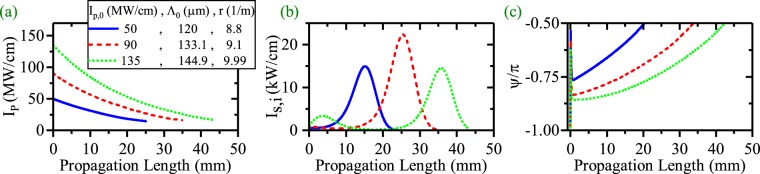
(**a**) Intensity variation of the HGM pump, (**b**) the SPP signal/idler generation and the SPP signal/idler propagation length enhancement [*λ*_*p*_ = 775 nm, *λ*_*s*_ = 1540 nm, *λ*_*i*_ = 1560.13 nm]. (**c**) Relative phase evolution (*ψ*) along the waveguide for the different values of *I*_*p*,0_ in ndDFG-CQPM case (*I*_*s*,0_ = 1 kW/cm and *I*_*i*,0_ = 0 kW/cm).

Another interesting way to mitigate the strong interaction between the HGM pump and the SPP modes is to use a chirped QPM (CQPM) domain grating^22^. The idea behind this method is that the pump cannot inject its power to the SPP modes at the beginning of the waveguide via changing the wavelength of domain inverted grating from its exact value $({{\rm{\Lambda }}}_{e}=\frac{2\pi }{{\rm{\Delta }}\beta })$. After that, the wavelength of domain inverted grating slowly reaches to the exact value when the waves propagate to the end of the waveguide^22^. This can be achieved with an inverse linearly chirped domain grating where its wavelength (Λ) varies according to equation 6:

6 $${\rm{\Lambda }}=\frac{{{\rm{\Lambda }}}_{0}}{1+rx},$$

where Λ_0_ and *r* are the wavelength of the CQPM grating at the beginning of the waveguide and chirping rate, respectively. The process to choose the Λ_0_ and *r* are completely described in the previous study^22^. This method enables us to mitigate strong interaction between the pump, signal and idler. Therefore, the HGM pump has enough power to compensate SPP modes attenuation for longer propagation lengths. These results were discussed in our previous work^22^ very concisely for the ndDFG based OPA interaction. Here a more detailed study of the PS ndDFG and dDFG based SPP length enhancement for CQPM waveguide is presented. Figure 9 presents the pump depletion, the SPP signal/idler generation (SPP length enhancement) and the relative phase evolution in panel (a), (b) and (c) respectively for ndDFG process in the CQPM waveguide (*λ*_*p*_ = 775 nm, *λ*_*s*_ = 1540 nm, *λ*_*i*_ = 1560.13 nm). In this case, *I*_*s*,0_ = 1 kw/cm and *I*_*i*,0_ = 0 kw/cm. The results show that enhancement of the *I*_*p*,0_ does not lead to a strong interaction between the HGM pump and the SPP signal and the SPP idler when the suitable chirping parameters (Λ_0_,*r*) are chosen (the related chirping parameters for each *I*_*p*,0_ are listed in Fig. 9(a)). Therefore the main obstacle to enhance the SPP propagation length is removed via CQPM technique. According to Fig. 9(b), when *I*_*p*,0_ = 50,90 and 135 MW/cm, the L-max is 22.4, 34.50 and 43.5 mm respectively. No sharp depletion in the pump variation and no high SPP amplification are seen. Figure 9(c) shows that *ψ* evolves along the propagation direction and for higher values of *I*_*p*,0_, it reaches to the stable value of $\frac{-\pi }{2}$ at longer distances. This is the main reason why the strong interaction between the HGM pump and the SPP modes stops.

**Figure 10:**
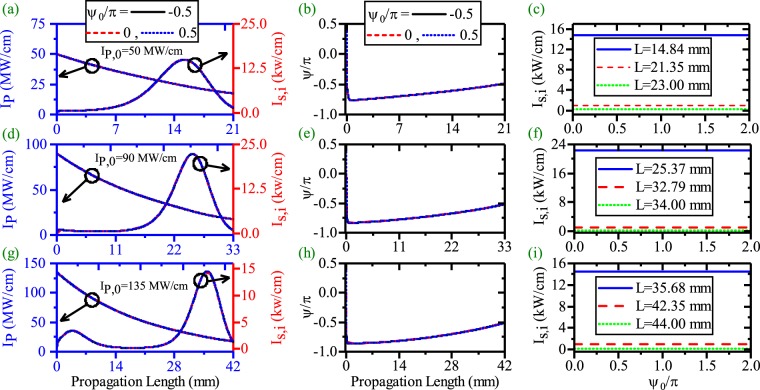
Effects of the *ψ*_0_ on the Pump depletion, the SPP signal/idler amplification and SPP signal/idler length enhancement in ndDFG-CQPM case (*I*_*s*,0_ = 1 kW/cm and *I*_*i*,0_ = 0 kW/cm). The HGM pump depletion (left-blue vertical axis) and the SPP signal/idler generation and propagation length enhancement (right-red vertical axis) along the propagation direction ((**a**),(**d**) and (**g**)). Relative phase (*ψ*) evolution along the propagation direction((**b**),(**e**) and (**h**)). Effects of *ψ*_0_ on the *I*_*s*,*i*_ generation at different positions ((**c**),(**f**) and (**i**)).

From the variation of *ψ*, it could be inferred that each change in *ψ*_0_ may cause a change in the evolutionary path, the pump depletion and the SPP propagation length enhancement. This is examined in Fig. 10.

**Figure 11:**
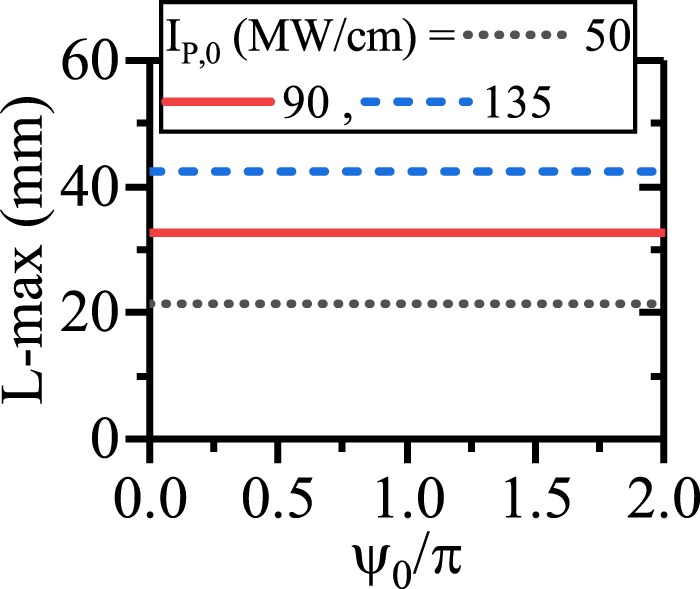
Maximum of the propagation length of the SPP signal/idler with respect to *ψ*_0_ for different values of *I*_*p*,0_ in ndDFG-CQPM case (*I*_*s*,0_ = 1 kW/cm and *I*_*i*,0_ = 0 kW/cm).

Panels (a),(d) and (g) present the pump depletion, SPP idler/signal generation and SPP length enhancement for different values of *ψ*_0_ and *I*_*p*,0_. The chirping parameters are the same as what reported in Fig. 9. According to these results, *ψ*_0_ does not affect on the SPP length enhancement despite *ψ* evolves along the CQPM waveguide. The evolution of *ψ* along the CQPM waveguide are depicted in panels (b),(e) and (h) for *I*_*p*,0_ = 50, 90 and 135 MW/cm respectively. These figures reveal that each evolutionary path drops after a very short propagation length to values around $-\frac{\pi }{2}$. This is because of the generation of non-phase-matched SPP idler just at the CQPM waveguide beginning. The difference from the stable value of $-\frac{\pi }{2}$ results in a low rate of the SPP idler generation at the waveguide beginning. With the propagation of the waves, *ψ* reaches to the stable value and the pump can transfer more power to the SPP signal and idler. Looking at the panels (a), (d) and (g), it can be seen that the major amplification of the SPP waves occurs at the end of the CQPM waveguide. The effect of *ψ*_0_ on the signal/idler generation are investigated in panels (c), (f) and (i). From the figures, it is inferred that the idler generation does not depend on *ψ*_0_. This independency holds for each *I*_*p*,0_ and each measurement length. As expected from the results, for the ndDFG interaction in the CQPM waveguide, *ψ*_0_ cannot affect the SPP signal/idler propagation length. To visualize this, effect of *ψ*_0_ on the SPP idler propagation length is shown in Fig. 11 for different values of *I*_*p*,0_ and reported chirping parameters in Fig. 9.

Consequently, for ndDFG in a CQPM waveguide, when *I*_*i*,0_ = 0 kw/cm, the only factors that results in a SPP length enhancement are *I*_*p*,0_ and the chirping parameters. For example when *I*_*p*,0_ = 135 MW/cm, Λ_0_ = 144.9 *μ*m and *r* = 9.99/m, the L-max of the SPP idler is 42.35 mm.

Up to this point, three methods leading to PS OPA of the SPP signal/idler were presented. Any combination of the mentioned methods may lead to a new evolutionary path and affect the pump depletion, the SPP waves generation and the SPP propagation length enhancement. At the rest of the paper the results of such studies are presented and compared.

**Figure 12:**
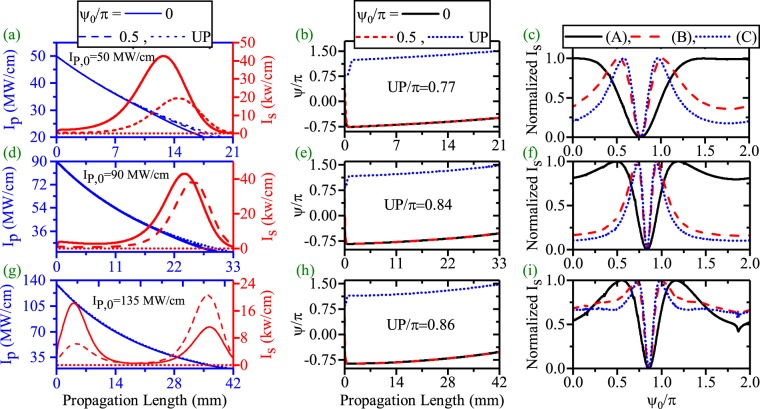
Effects of the *ψ*_0_ on the SPP signal amplification and SPP signal length enhancement in dDFG-CQPM case [*λ*_*p*_ = 775 nm, *λ*_*s*_ = 1550 nm]. The HGM pump and the SPP signal variations along the propagation direction ((**a**),(**d**) and (**g**). Relative phase (*ψ*) evolution along the propagation direction((**b**),(**e**) and (**h**). Phase sensitivity of the *I*_*s*_ at different position,[(**c**)-(A, L = 15.95 mm, *I*_*s*,*max*_ = 33.5 kw/cm),(B, L = 22 mm, *I*_*s*,*max*_ = 1 kw/cm) and (C, L = 25 mm, *I*_*s*,*max*_ = 0.03 kw/cm)], [(**f**)-(A, L = 26.97 mm, *I*_*s*,*max*_ = 34.5 kw/cm), (B, L = 34.4 mm, *I*_*s*,*max*_ = 1 kw/cm) and (C, L = 36 mm, *I*_*s*,*max*_ = 0.25 kw/cm)], [(**i**)-(A, L = 36.58 mm, *I*_*s*,*max*_ = 20.7 kw/cm), (B, L = 43.35 mm, *I*_*s*,*max*_ = 1 kw/cm) and (C, L = 45 mm, *I*_*s*,*max*_ = 0.18 kw/cm)].

Firstly, the effects of *ψ*_0_ on the SPP propagation length enhancement and the related results are investigated in the CQPM waveguide for the dDFG case. The pump and SPP signal variations along the propagation direction are shown in panels (a), (d) and (g) of Fig. 12 for *I*_*P*,0_ = 50 (Λ_0_ = 120 *μ*m, *r* = 8.7/m), 90 (Λ_0_ = 133.1 *μ*m, *r* = 9/m) and 135 MW/cm (Λ_0_ = 144.9 *μ*m, *r* = 9.89/m), respectively. As one can see, *ψ*_0_ affects the pump depletion, the SPP signal generation and the SPP propagation length enhancement efficiently for dDFG case in CQPM waveguide. A close look at the results reveals that the initial relative phase of $\frac{\pi }{2}$ is no longer the unsuitable phase (UP) to SSP amplification (see Fig. 12(c), (f) and (i)). Instead, UP depends on *I*_*p*,0_ and the chirping parameters for each pump intensity. The UP is 0.77 *π*, 0.84 *π* and 0.86 *π* radians for *I*_*P*,0_ = 50, 90 and 135 MW/cm, respectively. Panels (b), (e) and (h) show the evolutionary path for ${\psi }_{0}=\mathrm{0,}\frac{\pi }{2}$ and UP, respectively. With a comparison to the corresponding results in Figs 5 and 10, it is inferred that the resultant evolution of the phase from the CQPM grating is prior to the effects of *ψ*_0_ except for the UP case. On the other hand, because of low depletion of the pump intensity, the main mechanism to the pump depletion is the pump loss and around the maximum of the SPP propagation length, the pump intensity drops to the levels below its threshold. The evolution of *ψ* for different values of *ψ*_0_ and *I*_*p*,0_ are shown in panels (b), (e) and (h) of Fig. 12. According to the results, for *ψ*_0_ = 0, $\frac{\pi }{2}$, *ψ* reaches to values around the stable value of −$\frac{\pi }{2}$. This happens at the waveguide beginning which results in the SPP signal amplification. When the interacting waves go on along the CQPM waveguide, *ψ* achieves its stable value. In contrast, when *ψ*_0_ = UP, reaching to the stable value crosses a different path. In this case, *ψ* does not approach to the stable value at the beginning. Achieving the stable value happens when the pump intensity drops to the values below the threshold which results in no amplification of the SPP signal. The variation of the signal amplification with respect to the *ψ*_0_ variations is the subject of the panels (c), (f) and (i) of the Fig. 12 at different distances where case (A), (B) and (C) represent the peak of the *I*_*s*_ position, L-max and a bit more distant from the maximum of the SPP propagation length, respectively. Although, the interaction is in low depletion regime, no sin behavior is seen for case (A). The UP is pump dependent for CQPM case.

**Figure 13:**
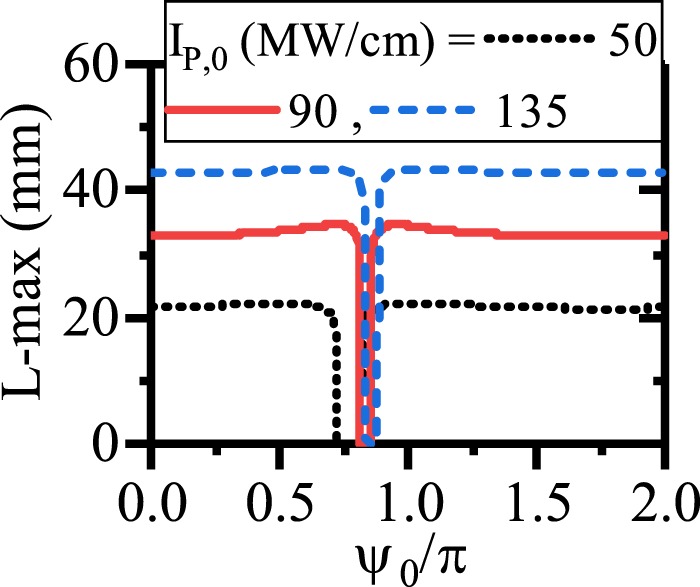
Maximum of the propagation length of SPP signal with respect to *ψ*_0_ for different values of *I*_*p*,0_ in dDFG-CQPM case.

To have a better insight on this subject, the effects of *ψ*_0_ on the enhancement of the SPP propagation length for different initial pump intensities and suitable values of chirping parameters are depicted in Fig. 13. Generally, from the figure it is inferred that an enhancement of the pump intensity in the CQPM waveguide could lead the SPP propagation length enhancement when the suitable values of the chirping are chosen. Using the UP with higher values of pump intensity for the CQPM case cannot lead to the enhancement of the SPP propagation length. The reason is that, around the maximum of the SPP propagation length, the pump intensity drops to the levels below its threshold and cannot contribute in the SPP propagation length enhancement for the CQPM waveguide. This result is in contrast to the results reported for Fig. 6. Therefore *ψ*_0_ cannot affect the SPP propagation length mainly except for a narrow band around the UP where the L-max drops to zero. The UP depends on the *I*_*p*,0_.

**Figure 14:**
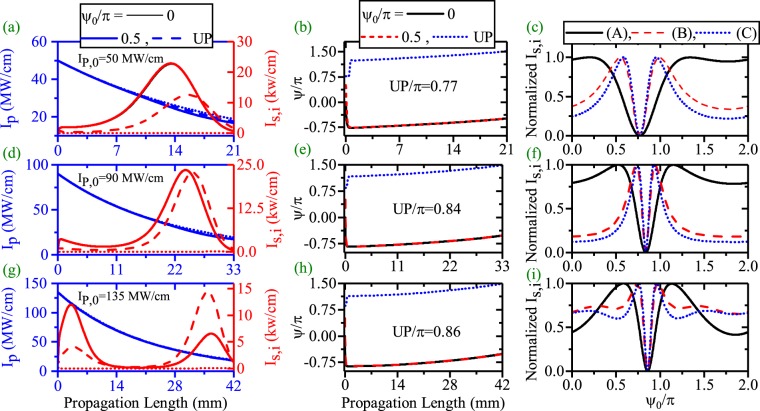
Effects of the *ψ*_0_ on the Pump depletion, the SPP signal/idler amplification and the SPP length enhancement in ndDFG-CQPM case (*I*_*i*,0_ = *I*_*s*,0_ = 1 kW/cm). The HGM pump depletion (left-blue vertical axis) and the SPP signal/idler generation and the SPP propagation length enhancement (right-red vertical axis) along the propagation direction ((**a**),(**d**) and (**g**)) [*λ*_*p*_ = 775 nm, *λ*_*s*_ = 1540 nm, *λ*_*i*_ = 1560.13 nm]. Relative phase (*ψ*) evolution along the propagation direction ((b), (e) and (h)). Phase sensitivity of the *I*_*s*_ at different position, [(**c**)-((A), L = 15.67 mm, *I*_*s*,*i*,*max*_ = 17.8 kw/cm), ((B), L = 21.52 mm, *I*_*s*,*i*,*max*_ = 1 kw/cm) and ((C), L = 23 mm, *I*_*s*,*i*,*max*_ = 0.22 kw/cm)], [(**f**)-((A), L = 26.88 mm, *I*_*s*,*i*,*max*_ = 19.9 kw/cm), ((B), L = 33.59 mm, *I*_*s*,*i*,*max*_ = 1 kw/cm) and ((C), L = 35 mm, *I*_*s*,*i*,*max*_ = 0.4 kw/cm)], [(**i**)-((A), L = 36.44 mm, *I*_*s*,*i*,*max*_ = 14.5 kw/cm), (B, L = 42.66 mm, *I*_*s*,*i*,*max*_ = 1 kw/cm) and (C, L = 44 mm, *I*_*s*,*i*,*max*_ = 0.3 kw/cm)].

The second combination for PS length enhancement is investigated when a light wave of angular frequency *ω*_*i*_ is injected at the input (*λ*_*p*_ = 775 nm, *λ*_*s*_ = 1540 nm and *λ*_*i*_ = 1560.13 nm, *I*_*s*,0_ = *I*_*i*,0_ = 1 kW/cm) of the CQPM waveguide. The pump and SPP signal/idler variations along the propagation direction are shown in panels (a), (d) and (g) of Fig. 14 for *I*_*P*,0_ = 50, 90 and 135 MW/cm respectively (The chirping parameters are the same as those reported in Fig. 9). The evolution of *ψ* along the propagation direction are depicted in panels (b), (e) and (h) of Fig. 14 for different values of *ψ*_0_. Panels (c), (f) and (i) of Fig. 14 show the generation of the signal/idler SPP modes at different distances which reported completely in the caption.

**Figure 15:**
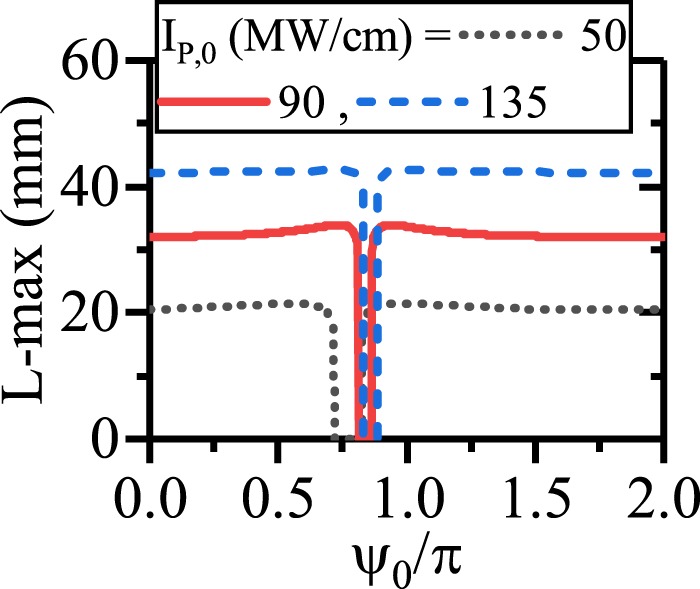
Maximum of the propagation length of the SPP signal/idler with respect to *ψ*_0_ for different values of *I*_*p*,0_ in ndDFG-CQPM case when a light wave of angular frequency *ω*_*i*_ is injected to the waveguide beginning (*I*_*i*,0_ = *I*_*s*,0_ = 1 kW/cm).

The obtained results are similar to the those reported in the dDFG interaction in the CQPM waveguide and the physics behind the SPP propagation length is the same as well. Therefore in this case, *ψ*_0_ can affect the SPP signal/idler generation but it cannot affect on the SPP propagation length enhancement intensively. The results section concludes with an illustration of the effects of *ψ*_0_ on the SPP signal/idler propagation length for the ndDFG case in the CQPM waveguide when a light wave of angular frequency *ω*_*i*_ is injected to the waveguide beginning. The results are shown in Fig. 15. Again, it can be seen that *ψ*_0_ has no role in the enhancement of the propagation length. Instead, around the UP which depends on the *I*_*p*,0_ and the related chirping parameters, L-max drops to zero. This is because at the corresponding L-max, the pump intensity drops to the values below the threshold level.

## Discussion

In summery, the adjustable SPP propagation length enhancement in a silver coated PPLN planar waveguide was investigated via phase sensitive difference frequency generation (DFG)-based optical parametric amplification (OPA). Different ways to create the position dependent relative phase between the HGM pump and the SPP modes (*ψ*(*x*)) were discussed. Utilizing them, three main methods to change the phase insensitive (PIS) DFG interaction for enhancing the SPP propagation length into phase sensitive (PS) phenomenon were presented. These three main methods are :1) Using the dDFG interaction instead of ndDFG case. 2) Inserting a light wave of angular frequency *ω*_*i*_ at the waveguide beginning. 3) Using a chirped QPM (CQPM) waveguide to mitigate the strong interaction between the interacting waves and create a position dependent relative phase. The results show that:

- ndDFG interaction is a PIS phenomenon in the EQPM waveguide. Initial relative phase (*ψ*_0_) cannot affect the SPP signal/idler generation and the SPP propagation length enhancement. The main method to enhance the SPP propagation length is to use a suitable value of *I*_*p*,0_. for example when *I*_*p*,0_ = 31 MW/cm, the SPP propagation length is about 11.5 mm. *I*_*p*,0_ values above this value result in a strong interaction and shortening the the SPP propagation length.

- dDFG interaction is PS in the EQPM waveguide. In this case, different values of *ψ*_0_ lead to shortening or enhancing the SPP propagation length. The unsuitable phase (UP) to enhance the SPP propagation length is $\frac{\pi }{2}$. For low values of *I*_*p*,0_ this UP results in no amplification of the SPP wave. To remove this obstacle, higher values of *I*_*p*,0_ is needed. For instance, when *I*_*p*,0_ = 66 MW/cm and ${\psi }_{0}=\frac{\pi }{2}$ the SPP propagation length reaches to about 30 mm.
- Inserting a light wave at the angular frequency of *ω*_*i*_ in the input of the EQPM waveguide leads to a PS manner. In this case, manipulation of *ψ*_0_ can enhance the SPP propagation length. According to the results for *I*_*p*,0_ = 33 MW/cm and ${\psi }_{0}=\frac{\pi }{2}$, the SPP propagation length reaches to about 16 mm.
- Using the CQPM waveguide is another way to manipulate the evolution of *ψ* along the waveguide. In this way, for ndDFG case the SPP propagation length reaches to 43 mm when *I*_*p*,0_ = 135 MW/cm. But because of the generation of the SPP idler at the waveguide beginning, the overall process is insensitive to *ψ*_0_. Therefore reaching to the SPP propagation length up to 43 mm could be possible without any knowledge about *ψ*_0_.
- dDFG interaction in the CQPM waveguide is sensitive to *ψ*_0_. For this case, the resultant evolution of *ψ* from the CQPM waveguide is prior to the resultant variation of *ψ* from the dDFG interaction. The UP is pump dependent and differs from $\frac{\pi }{2}$. The results show that around the UP, the SPP propagation length drops to zero. Increasing of *I*_*p*,0_ cannot enhance the SPP propagation length around UP.
- The results for the ndDFG interaction with inserting a light wave of angular frequency *ω*_*i*_ at the beginning of the CQPM waveguide are very similar to the dDFG case in the CQPM waveguide and the justifying physics is the same.

In conclusion, the phase sensitive DFG-based OPA leads to the adjustable propagation length enhancement of the SPP signal. The controlling variable then would be the input idler and HGM pump phase for ndDFG and the HGM pump phase for the dDFG one. The Authors believe that the phase-sensitive OPA may help coherent amplification (or deamplification) of the SPPs in the field of quantum plasmonics where the adjustable SPP propagation length or phase-sensitive OPA interaction are needed^25^. Specially this study can be used in the study on nonlinear interactions in the coaxial plasmonic waveguides which have transverse dimensions of the order of few hundred nanometers^26^.
